# Hypersensitivity in teeth affected by molar-incisor hypomineralization (MIH)

**DOI:** 10.1038/s41598-021-95875-x

**Published:** 2021-09-09

**Authors:** Thomas Linner, Yeganeh Khazaei, Katharina Bücher, Jan Pfisterer, Reinhard Hickel, Jan Kühnisch

**Affiliations:** 1 Department of Conservative Dentistry and Periodontology, University Hospital, Ludwig-Maximilians University of Munich, Munich, Germany; 2 Poliklinik für Zahnerhaltung und Parodontologie, Klinikum der Universität München, LMU München, Goethestraße 70, 80336 Munich, Germany

**Keywords:** Dental diseases, Dental caries

## Abstract

Tooth hypersensitivity is a common symptom in patients with molar-incisor hypomineralization (MIH). Therefore, this clinical study aimed to assess potential associations between patient- and tooth-related variables and the intensity of hypersensitivity in MIH-affected permanent teeth compared to healthy controls. Fifty-seven MIH patients and 20 healthy adolescents with a total of 350 MIH-affected and 193 healthy teeth were included in this study. The intensity of hypersensitivity was measured after cold air stimulation using the Schiff Cold Air Sensitivity Scale (SCASS) by the dentist and visual analogue scale (VAS) by the patient. Tooth hypersensitivity was low in non-MIH teeth (97.9% of the group had zero SCASS and VAS values). In contrast, MIH-affected teeth with demarcated opacities and atypical restorations had moderate SCASS and VAS values, whereas teeth with enamel breakdown were mostly linked to severe hypersensitivity. The logistic regression model confirmed a significantly lower level of hypersensitivity in MIH patients aged ≥ 8 years (OR 0.06, 95% CI 0.01–0.50, p = 0.009) and higher levels in molar teeth (OR 5.49, 95% CI 1.42–21.27, p = 0.014) and teeth with enamel disintegration (OR 4.61, 95% CI 1.68–12.63, p = 0.003). These results indicate that MIH-related tooth hypersensitivity seems to be present in disintegrated molars immediately after tooth eruption.

## Introduction

Tooth hypersensitivity to thermal or mechanical stimuli is a common clinical symptom in patients with molar-incisor hypomineralization (MIH) and is a frequent reason to consult a dentist^1^. According to the model by Brännström^2^, dentinal hypersensitivity is caused by exposed dentine and disturbances in fluid-filled dentinal tubules after thermal, chemical, tactile or osmotic changes^3,4^. Movement of dentinal fluid stimulates baroreceptors, which lead to neural discharge and the sensation of pain^5^. In the case of MIH, the affected enamel is characterized by a reduction in mineral quantity and quality as well as an increased porosity^6^ and might therefore exhibit reduced thermal isolation properties and altered thermal conductivity properties. Furthermore, reduced hardness and elastic modulus, increased carbon and carbonate concentrations and a higher protein content compared to normal human enamel have been described^7–12^, leading to posteruptive enamel breakdown and the early exposure of porous subsurface enamel or dentine^13–16^. As a clinical consequence, the increased sensitivity leads to a deterioration of oral hygiene with increased plaque accumulation, which finally results in a higher susceptibility to caries^17,18^. While hypersensitivity is one of the main symptoms in children with MIH^13,19,20^, it is surprising that only two author groups have published scientific data on this issue thus far^18,21^. Both groups confirmed the common clinical observation of marked hypersensitivity and reported a proportional increase with increasing MIH severity. To date, no information is available about the influence of relevant patient-related (age, sex) and tooth-related (MIH severity, defect size, type of tooth) variables on hypersensitivity in MIH-affected teeth. Therefore, this clinical study aimed to investigate the potential influence of these variables on the severity of MIH-related tooth hypersensitivity. The null hypothesis was that there is no difference in hypersensitivity in MIH-affected teeth and healthy teeth.

## Materials and methods

This clinical study was conducted in accordance with the Declaration of Helsinki and was reported following the Strengthening the Reporting of Observational studies in Epidemiology (STROBE) guidelines for observational studies^22^. Ethics approval was obtained from the Human Ethics Committee of the medical faculty of Ludwig-Maximilians-University, Munich on June 18, 2018 (Project no. 18-249). Written informed consent was obtained from all children and their parents/legal guardians before study participation.

### Patient sample

The patients included in the study were between 6 and 18 years of age and had at least one MIH-affected tooth presenting an enamel breakdown or atypical restoration. Individuals with fluorosis; genetic disorders, such as amelogenesis/dentinogenesis imperfecta; or enamel defects other than MIH were excluded from this investigation. Before conducting the study, a sample size calculation was performed considering different scenarios (N = 50, power = 79%, effect size d = 0.7; N = 75, power = 91%, effect size d = 0.7; N = 100, power = 97%, effect size d = 0.7). Subsequently, the study population was recruited as follows. In the first step, the patient documentation system at the Department of Conservative Dentistry and Periodontology was used to screen for subjects who visited the clinic with a potential MIH diagnosis between February 2010 and December 2018. A total of 377 MIH patients were primarily identified. Of this population, 259 children visited the clinic once or only sporadically. A total of 118 individuals had regular dental check-ups and were treated operatively due to MIH-related dental defects. The latter group was invited to participate in the study, and 57 individuals ultimately agreed. In addition, 20 children with dentition free of MIH, caries and other dental defects served as controls. For this convenience sample, an alpha of 5%, a confidence interval of 95% and a difference of 0.7 between two independent means (two groups) yields a power of 0.85.

At the time of clinical evaluation, the mean and median age of the MIH group (N = 57; 26 females/31 males) were 10.9 years and 10.0 years, respectively (standard deviation (SD) = 2.9, Min = 6.6, Max = 18.2). These parameters were 11.6 years and 12.0 years (SD = 3.8, Min = 6.8, Max = 18.0), respectively, in the control group (N = 20; 10 females/10 males).

### Clinical examination

Standardized examination was performed for all included patients in a professional setting using a dental unit with an operation light. At the beginning of each clinical examination, professional tooth cleaning using a rotary brush (Hawe Miniature Brush, Nylon bristles, KerrHawe SA, Bioggio, Switzerland) and polishing paste (Proxyt medium, Ivoclar Vivadent GmbH, Ellwangen, Germany) was performed. Subsequently, a trained and calibrated dentist (TL) conducted all examinations of each tooth and surface using standard conditions and a plane dental mirror (HS-Maxima, Mouthmirrors Rhodium Nr. 4, plane, Henry Schein Dental Deutschland GmbH, Langen, Germany), a blunt Community Periodontal Index (CPI) probe (CP-11.5B6, Hu-Friedy, Chicago, IL, USA) and a dental syringe (3F Syringe, KaVo, Biberach, Germany). Caries status was determined by recording the Decayed, Missing, Filled (DMF) index^23^ as well as non-cavitated caries lesions (NCCLs) according to the International Caries Detection and Assessment System (ICDAS)/Universal Visual Scoring System (UniViSS) criteria^24,25^. No additional bitewing radiographs were performed. MIH-related demarcated opacities, enamel breakdowns, atypical restorations and extractions were diagnosed according to the European Academy of Paediatric Dentistry (EAPD) criteria for all permanent teeth and surfaces^13^ because MIH can be found not only on defined index teeth but also on all teeth^26^. Demarcated opacities with a diameter < 1 mm were not recorded. MIH-associated defects or restorations were not scored with the DMF index. In the case of multiple findings on a tooth or surface, caries and MIH were recorded separately.

The intensity of tooth hypersensitivity was measured by stimulating the buccal surface of a tooth with air from a dental syringe (3.3 bar at room temperature) at a distance of approximately 1 cm for 1 s with adjacent teeth shielded by the examiner’s fingers. The Schiff Cold Air Sensitivity Scale (SCASS)^27^ was used by the examiner to assess the subjects’ response to this stimulus. Scoring was performed as follows: 0 = subject does not respond to air stimulus; 1 = subject responds to air stimulus but does not request discontinuation of stimulus; 2 = subject responds to air stimulus and requests discontinuation or moves away from stimulus; and 3 = subject responds to air stimulus, considers stimulus to be painful, and requests discontinuation of the stimulus. In addition, the patient was asked to estimate the current level of pain perceived directly after air stimulation using a visual analogue scale (VAS), which is based on the Wong-Baker Faces Pain Rating Scale^28^ and ranges from 0 (no pain) to 10 (the worst pain). VAS values were rated as follows: mild (0–2), moderate (3–4) and severe (5–10) according to Gerbershagen et al.^29^. In MIH patients, hypersensitivity testing was performed on all MIH-affected teeth and in healthy patients on all first permanent molars and six randomly selected permanent incisors. In total, 543 anterior (N = 285) and posterior permanent teeth (N = 258) were available for hypersensitivity testing.

### Calibration before examination

Before the study, two-day calibration training was undertaken with the examiner (TL) by an experienced clinician and epidemiologist (JK) who provided information about the study design, indices and diagnostic principles. After theoretical training with clinical photographs, practical training for clinical data collection was performed on 10 patients who were not included in the study. At the end of the calibration training, the examiner evaluated one hundred twenty unknown, high-quality photographs from occlusal and smooth surfaces for the detection of cavitated carious lesions and caries-associated restorations (DMF index), NCCLs and MIH. Detailed information on kappa values is presented in Table 1.

**Table 1:** Results from the calibration phase for inter- and intraexaminer reproducibility (unweighted kappa values).

	Occlusal surfaces			Smooth surfaces		
	DMF	UniViSS	MIH	DMF	UniViSS	MIH
TL (intra)	0.873	0.901	0.950	0.932	0.851	0.962
TL (inter)	0.934	0.963	0.975	0.932	0.903	0.869

### Data management and statistics

An electronic case report form (EpiData, EpiData Association, Odense, Denmark, version V4.4.1.0) was designed to facilitate the structured data entry of relevant information about patient characteristics, caries, MIH status and hypersensitivity. In the next step, the compiled EpiData database was exported to Excel (Office 2016 Excel, Microsoft Corporation, Redmond, WA, USA) for further processing. Statistical analysis was undertaken using R software (version 3.6.0, R Development Core Team, Vienna, Austria). The significance level was set at α = 0.05, with a 95% confidence interval. As each person has more than one tooth, data collected and analysed at the tooth level are likely to be correlated within individuals. Therefore, a mixed logistic regression model^30,31^ with the statistical software package lme4 (version 1.1-21) was used to account for the clustered nature of the data and to safely fit the investigated variables (age in years, sex, type of tooth, defect size, MIH score) in the existing model based on the population size of teeth/patients^32,33^. Furthermore, we observed a high number of zero values for both measures of hypersensitivity (VAS and SCASS); hence, both variables were reconfigured into binary categorical variables indicating the presence or absence of sensitivity for use in the mixed logistic regression model^34,35^. Spearman’s rank correlation was used for the correlation analysis between the dentist’s and patients’ hypersensitivity assessments. The statistical software package ggplot2 (version 3.3.0) was used to create the plots and trendlines. In the mixed logistic regression model, ten events can be assumed for each variable/cluster. Therefore, five variables (age in years, sex, type of tooth, defect size, MIH score) can be safely fit in the logistic regression model^32,33^.

## Results

The characteristics of the study population are shown in Table 2. In the MIH group, a mean number of 6.6 (SD = 3.8) MIH-affected permanent teeth was found. A total of 54.6% of the MIH lesions were registered on permanent molars (N = 192) and premolars (N = 14), and 45.4% were identified on incisors (N = 153) and canines (N = 18).

**Table 2:** Characterization of the dental status in the permanent dentition of both study groups.

		MIH group		Control group	
		Mean (SD)	%	Mean (SD)	%
Caries	**Teeth with non-cavitated caries lesions**	0.9 (2.2)	90.0	1.4 (3.4)	93.3
	**DMF/T**	0.1 (0.4)	10.0	0.1 (0.2)	6.7
	Teeth with cavitated caries lesions (D/T)	0.0 (0.1)	0.0	0.0 (0.0)	0.0
	Restored teeth due to caries (F/T)	0.1 (0.3)	10.0	0.1 (0.2)	6.7
	Extracted teeth due to caries (M/T)	0.0 (0.0)	0.0	0.0 (0.0)	0.0
	Total	1.0 (2.4)	100.0	1.5 (3.5)	100.0
**MIH**	**Teeth with demarcated opacities**	3.4 (2.2)	51.5	–	–
	Molars	1.0 (1.0)	15.1		
	Incisors	2.4 (2.0)	36.4		
	**Teeth with enamel disintegrations**	0.9 (1.1)	13.6	–	–
	Molars	0.8 (1.1)	12.1		
	Incisors	0.1 (0.4)	1.5		
	**Teeth with atypical restorations**	2.1 (2.8)	31.9	–	–
	Molars	1.6 (2.0)	24.3		
	Incisors	0.5 (1.4)	7.6		
	**Extracted teeth due to MIH**	0.2 (1.1)	3.0	–	–
	Molars	0.2 (1.1)	3.0		
	Incisors	0.0 (0.0)	0.0		
	Total	6.6 (3.8)	100.0	–	–

**Figure 1:**
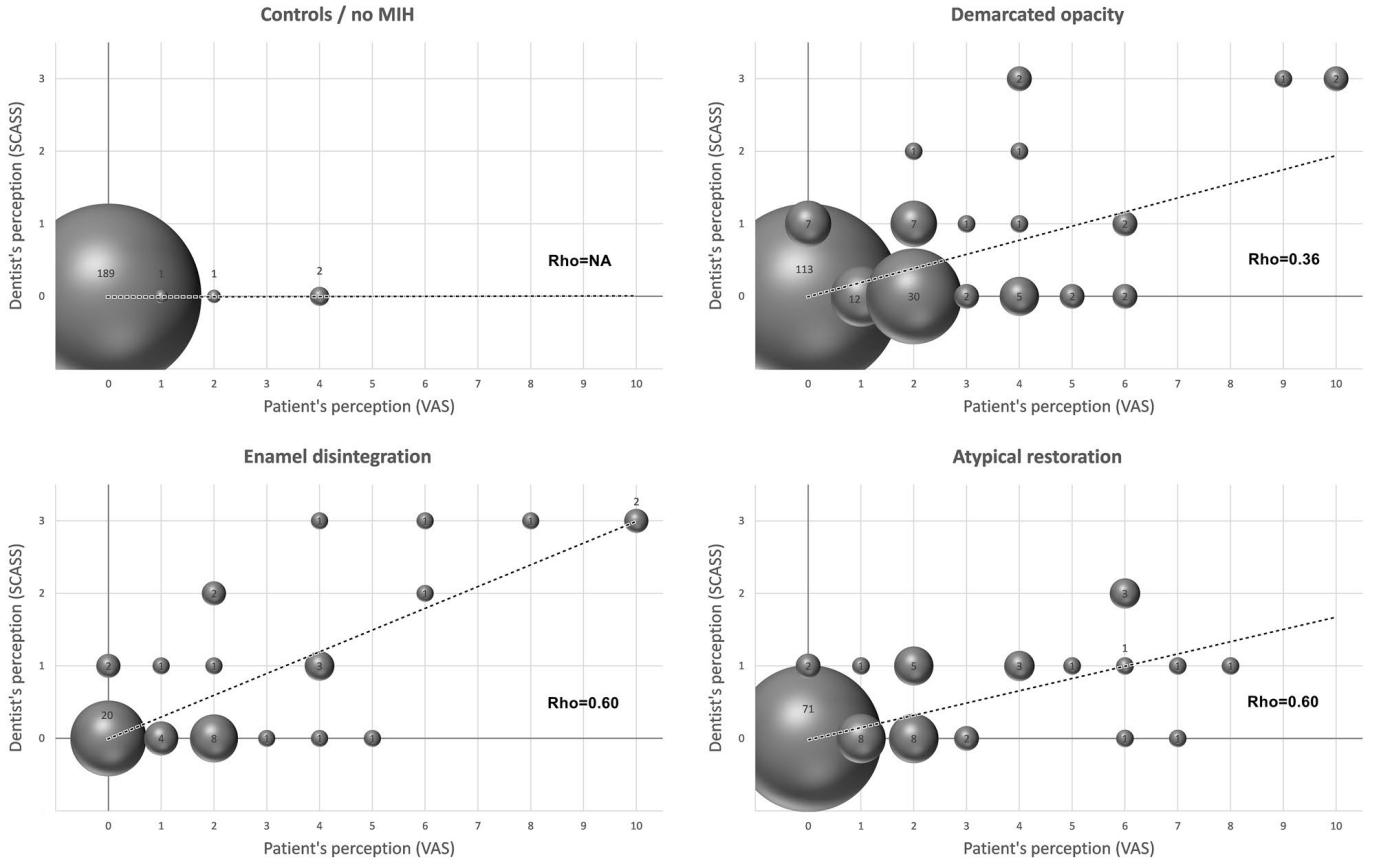
Spearman’s rank correlation between the perceptions of the dentist (SCASS) and the patient (VAS).

**Figure 2:**
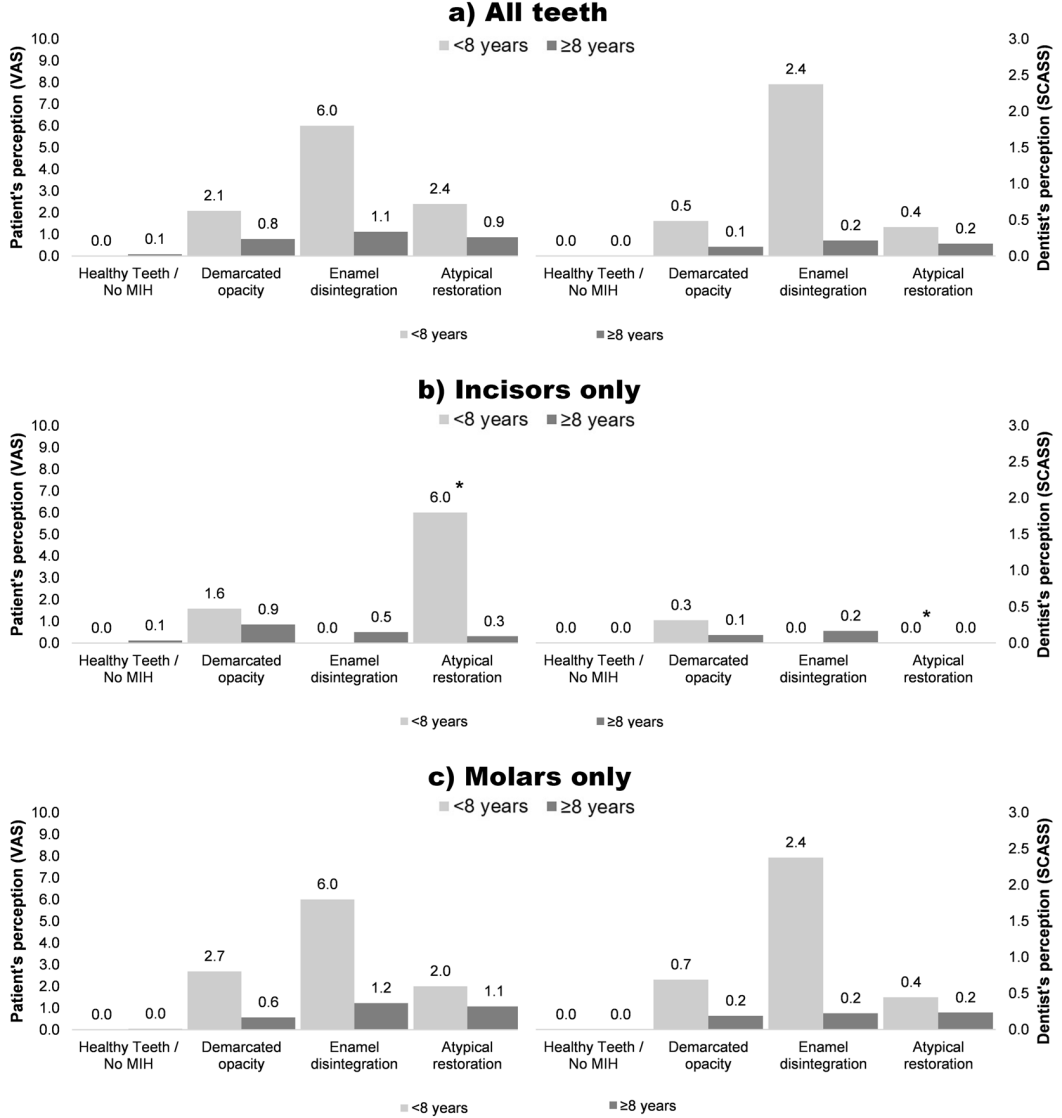
MIH-related hypersensitivity plotted according to the type of tooth in younger (< 8 years) and older (≥ 8 years) children. *Mean value is based on data from only one patient.

**Figure 3:**
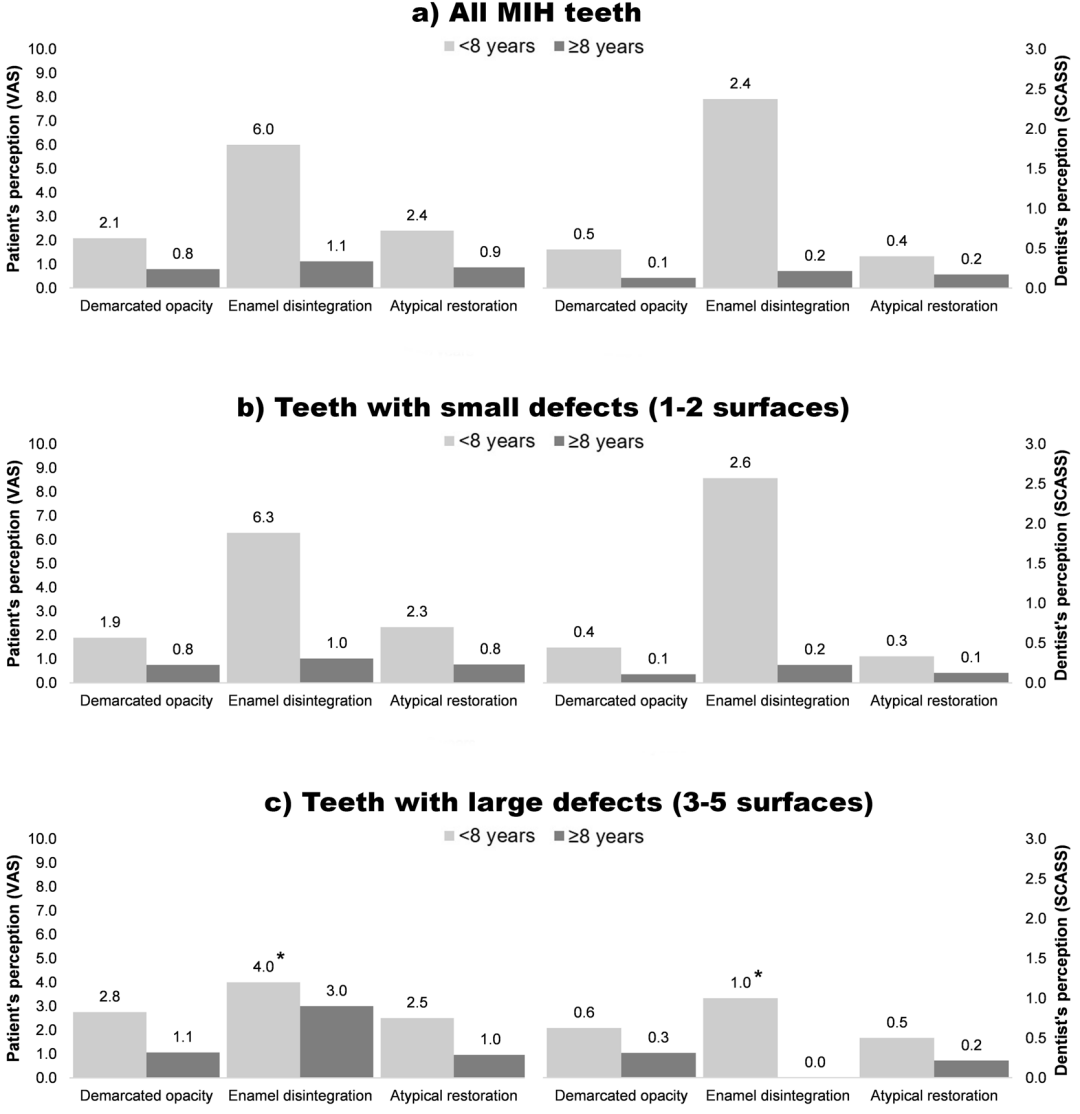
MIH-related hypersensitivity plotted according to the size of the MIH lesions in younger (< 8 years) and older (≥ 8 years) children. *Mean value is based on data from only one patient.

A total of 350 MIH-affected teeth (demarcated opacity: N = 191, enamel disintegration: N = 50, atypical restoration: N = 109) and 193 healthy teeth were available for hypersensitivity testing. Since both applied scales are based on a subjective assessment by either the patient (VAS) or the dentist (SCASS), we compared their consistency with Spearman’s rank correlation and found good agreement between both scales (Fig. 1). When comparing the intensity of hypersensitivity, low hypersensitivity values (VAS and SCASS) were found in non-MIH teeth (controls), with 97.9% zero values. MIH-affected teeth with demarcated opacities and atypical restorations had mild to moderate hypersensitivity, whereas teeth with enamel disintegration were mostly linked to severe hypersensitivity. Younger patients—especially those under the age of 8 years—rated the level of discomfort or pain higher than older children after air blast stimulation (Figs. 2, 3). Furthermore, molars seem to be more prone to hypersensitivity than incisors independent of their MIH score (Fig. 2), whereas the defect size did not seem to influence hypersensitivity (Fig. 3). The abovementioned associations were also confirmed in the logistic regression model (Table 3). Here, hypersensitivity was significantly higher in MIH-affected teeth in patients younger than 8 years, MIH-affected molars and MIH-affected teeth with enamel disintegration, whereas sex and defect size did not have a significant effect.

**Table 3:** Results from the mixed logistic regression model between hypersensitivity perceived by MIH patients (Visual Analogue Scale, range 0–10) and the dentist (Schiff Cold Air Sensitivity Scale, range 0–3) and potential influencing variables. Significant values are reported in bold font.

	VAS		SCASS	
	Estimate (95% CI)	p-value	Estimate (95% CI)	p-value
**Age in years**				
< 8 (N = 53)	1.00		1.00	
≥ 8 (N = 297)	0.36 (0.11–1.24)	0.106	**0.06 (0.01–0.50)**	**0.009**
**Sex**				
Male (N = 171)	1.00		1.00	
Female (N = 179)	1.22 (0.47–3.17)	0.688	3.58 (0.62–20.50)	0.152
**Type of tooth**				
Incisor (N = 171)	1.00		1.00	
Molar (N = 179)	1.21 (0.29–1.39)	0.257	**5.49 (1.42–21.27)**	**0.014**
**Defect size**				
Small (1–2 MIH-affected surfaces) (N = 268)	1.00		1.00	
Large (3–5 MIH-affected surfaces) (N = 82)	1.78 (0.78–4.08)	0.172	1.20 (0.54–2.68)	0.647
**MIH score**				
Demarcated opacity (N = 191)	1.00		1.00	
Enamel disintegration (N = 50)	**4.61 (1.68–12.63)**	**0.003**	1.52 (0.33–7.00)	0.593
Atypical restoration (N = 109)	1.41 (0.62–3.20)	0.407	1.27 (0.37–4.32)	0.698

## Discussion

This clinical study analysed the intensity of hypersensitivity in patients with MIH-affected teeth with demarcated opacities, enamel disintegration or atypical restorations in comparison to healthy teeth. When comparing data on hypersensitivity, the initial null hypothesis has to be rejected because MIH was associated with more sensitive teeth than healthy teeth (Table 3, Figs. 2, 3).

In general, this study confirms the problem of tooth hypersensitivity in MIH patients, and potential influencing factors, e.g., MIH score, age, sex, tooth type and defect size, were considered. The data showed that intact MIH teeth—typically scored as demarcated opacities—are less sensitive than teeth with enamel disintegration (Table 3, Figs. 2, 3). Interestingly, teeth that were restored to cover the defective surfaces showed hypersensitivity, which is comparable to teeth with intact demarcated opacities (Table 3, Figs. 2, 3). This finding is consistent with data recently published by Fütterer et al.^36^, who showed that severely damaged teeth were not completely symptom free after the treatment of choice but that the restorative intervention reduced hypersensitivity and improved the quality of life of affected children. Contrary to this finding, the results from a Brazilian study indicated the presence of a substantial degree of hypersensitivity in restored MIH teeth^21^, which might be explained by different restorative procedures and a high percentage of additional dentine caries lesions. Based on the existing data, it might be argued that children suffering from MIH-related dental hard tissue defects may benefit from dental restoration to reduce hypersensitivity. Nevertheless, the differences between the studies might also be attributed to methodological discrepancies or the chosen samples^21,36^.

This study further confirms that younger patients suffer from a severe intensity of hypersensitivity, especially on their first permanent MIH molars immediately after tooth eruption. With increasing patient age, the clinical problem of tooth hypersensitivity consistently decreases (Table 3). Therefore, it can be concluded that MIH-related tooth hypersensitivity is most likely linked to the few years immediately after tooth eruption. Of course, individual deviations might be possible, but the data presented herein confirm this overall trend. Explanations for the decreasing hypersensitivity over time could be related to the processes of physiological dentine formation and reactive dentine apposition when the tubular dentin is exposed, which might furthermore be supported by regular topical fluoridation^13^, casein phosphopeptide-amorphous calcium phosphate (CPP-ACP) application^37^ or desensitizing products containing 8% arginine^38^. Another explanation might be that older children might exhibit an altered awareness of tooth hypersensitivity. Another finding from our data is that MIH-affected permanent molar teeth are significantly more hypersensitive than incisors, which might be caused by the fact that hard tissue breakdown is more frequently found on occlusion-bearing surfaces. However, the size of the MIH defect surprisingly had no significant effect on tooth hypersensitivity, which highlights the fact that the quality of mineralization, that is, the degree of hypomineralization, seems to be the more influential clinical variable.

The present study has strengths and limitations. One major strength of this study is that the chosen study design and logistic regression analyses allowed the investigation of the potential influence of different variables on MIH-related tooth hypersensitivity. Here, it should be mentioned that the present study included standard protocols for recording MIH defects^13^, provoking/recording hypersensitivity (SCASS) and documenting the patient´s pain perception (VAS). Furthermore, all hypersensitivity tests were performed by the same dentist. When considering the overall recorded descriptive and explorative data, it can be argued that there was, in general, acceptable agreement between the SCASS and VAS (Fig. 1). Nevertheless, it should not be ignored that the individual perception of pain, especially by young children, may have varied, resulting in a few outliers, which probably further influenced the outcomes of the mixed logistic regression model. This fact and the divergent number of categories between the SCASS and VAS may explain why significance varied between both tests in the mixed logistic regression model.

There are some limitations that need to be highlighted. Regarding the sample size, which initially appears small, it is important to note that the investigated group is composed of children with a severe MIH burden. When investigating MIH-related hypersensitivity, this is the group that needs to be included, as these children suffer the most from the problem of hypersensitivity. In epidemiological studies, this group represents ~ 10% of the overall group of MIH children^26,39,40^, which largely explains the small sample size and why it could not be substantially increased in a single-centre study. On the one hand, the low proportion of severely affected MIH patients resulted in the heterogeneous age of the included children and adolescents, which ranged from 6 to 18 years. In addition, the control group of healthy individuals was more homogenous. As described by Wacholder et al.^41^, the control group should represent the pool of patients from whence the cases are drawn and not the general population of the cases. Hence, given sufficient power, the sampling procedure from the same source population is fit to the given circumstances as long as we follow appropriate sample selection processes. Logistic regression and confounding adjustment were applied in the usual manner. On the other hand, this age distribution allowed analysis of a possible influence of age on the development of hypersensitivity. Nevertheless, the data should be interpreted with caution due to the small number of includable cases and limited representativeness. To overcome this problem, an adequately powered and representative epidemiological study is needed, which has not been conducted thus far. Furthermore, the group allocation method used needs to be further discussed. In the present investigation, we included a separate group of children who were not suffering from MIH, which resulted in independent controls. Contrary to this design, other studies measured hypersensitivity in unaffected molars of children suffering from MIH^18^. With respect to the observation that children with MIH might generally be negatively influenced by hypersensitive teeth, unbiased reporting from non-affected teeth in children with MIH could not be fully expected. Therefore, such recordings seem not to be independent. From a practical point of view, which is especially relevant for the present population, unaffected MIH molars were a rare finding. Therefore, we decided to form an independent control group. Furthermore, it should probably be taken into account that different restorative procedures or materials might be linked to divergent outcomes in terms of MIH-related tooth hypersensitivity. Therefore, future studies should be conducted to close this knowledge gap.

## Conclusion

In conclusion, tooth hypersensitivity is a serious clinical problem in children affected by MIH, and the degree of hypersensitivity is significantly higher in individuals aged ≤ 8 years, molar teeth and teeth affected by hard tissue breakdowns.
